# Complete Genome Sequence of the Rhizobacterium *Pseudomonas trivialis* Strain IHBB745 with Multiple Plant Growth-Promoting Activities and Tolerance to Desiccation and Alkalinity

**DOI:** 10.1128/genomeA.00943-15

**Published:** 2015-09-03

**Authors:** Arvind Gulati, Mohit Kumar Swarnkar, Pratibha Vyas, Praveen Rahi, Rishu Thakur, Namika Thakur, Anil Kumar Singh

**Affiliations:** a CSIR-Institute of Himalayan Bioresource Technology, Palampur, India; b Lovely Professional University, Phagwara, India; c National Centre for Cell Science, Pune, India

## Abstract

The complete genome sequence of 6.45 Mb is reported here for *Pseudomonas trivialis* strain IHBB745 (MTCC 5336), which is an efficient, stress-tolerant, and broad-spectrum plant growth-promoting rhizobacterium. The gene-coding clusters predicted the genes for phosphate solubilization, siderophore production, 1-aminocyclopropane-1-carboxylate (ACC) deaminase activity, indole-3-acetic acid (IAA) production, and stress response.

## GENOME ANNOUNCEMENT

The genus *Pseudomonas* belongs to the Gamma subclass of *Proteobacteria* (1) with 218 species (http://www.bacterio.net/pseudomonas.html). The fluorescent *Pseudomonas* have received attention due to their widespread distribution and predominance among the rhizobacteria, and versatility for improving plant growth and productivity (2, 3). *Pseudomonas trivialis* IHBB745, isolated from the rhizosphere of *Hippophae rhamnoides* growing in the Lahaul-Spiti district, India (31°45′ 33°15″ N 76°21′ 78°40″ E), has been reported as a phosphate solubilizer with high abiotic stress tolerance (4–6). The strain also possesses the attributes of indole-3-acetic acid (IAA) production, 1-aminocyclopropane-1-carboxylate (ACC) deaminase activity, and siderophore production. The complete genome sequence is reported to provide genomic information on the multifunctional plant growth-promoting activities of the strain.

The genomic DNA was isolated using a phenol-chloroform-isoamyl alcohol extraction procedure (7) from 24-h-old cultures. The genomic DNA was quantified by a NanoDrop 2000 UV-Vis spectrophotometer (Thermo Scientific, USA) and Qubit version 2.0 fluorometer (Invitrogen, USA). The genomic DNA was sheared using Covaris g-TUBEs, and quality was checked with a Bioanalyzer DNA 12000 chip (Agilent Technologies, USA). A PacBio SMRTbell library preparation kit version 1.0 was used per the manufacturer’s instructions to prepare the genomic DNA library with a DNA insert size of around 10 kb. The SMRTbell library quantified using a Qubit version 2.0 fluorometer was sequenced on the PacBio RS II system on three SMRT cells, employing a P5 polymerase and C3 chemistry combination (P5-C3) with 180-min movie. The SMRT cells produced 1,259,528,544 bases generated through 295,525 reads (*N*_50_ size 5,735 and mean subread length 4,262) (8). The subreads were assembled *de novo* using the RS Hierarchical Genome Assembly Process (HGAP) protocol version 2.0 in SMRT Analysis version 2.2.0 (Pacific Biosciences, USA). The functional annotations were performed on the Rapid Annotations using Subsystems Technology (RAST) server (9).

The complete circular genome of strain 745 was 6,452,803 bp in size with an estimated GC content of 59.91%. A total of 6,032 protein-coding genes and 84 RNA genes (9) were predicted in the genome. Based on the 16S rRNA gene sequences, the strain IHBB745 is closely related to *Pseudomonas trivialis* DSM 14937^T^ and *P. poae* DSM 14936^T^. The pairwise calculation of the average nucleotide identity using EzBiocloud (http://www.ezbiocloud.net) indicated 86.2% similarity between the genome sequences of strain IHBB745 and *Pseudomonas poae* RE1-1-14, confirming that the strains belonged to the different species. The annotation predicted gene-coding clusters for phosphate solubilization (6 genes), auxin biosynthesis (4 genes), siderophore production (31 genes), ACC deaminase activity (1 gene), HCN production (7 genes), and ammonia production (2 genes), attesting its multiple plant growth-promoting attribute. The stress tolerance trait of the strain also predicted 205 stress response genes related to osmotic stress (37 genes), oxidative stress (96 genes), cold shock (7 genes), heat shock (16 genes), peripalsmic stress (6 genes), and other stress responses.

### Nucleotide sequence accession number. 

The genome sequence of *Pseudomonas trivialis* strain IHBB745 has been deposited in GenBank under the accession number CP011507.
